# Automatic Generation of Validated Specific Epitope Sets

**DOI:** 10.1155/2015/763461

**Published:** 2015-10-19

**Authors:** Sebastian Carrasco Pro, John Sidney, Sinu Paul, Cecilia Lindestam Arlehamn, Daniela Weiskopf, Bjoern Peters, Alessandro Sette

**Affiliations:** ^1^Laboratorio de Bioinformática y Biología Molecular, Laboratorios de Investigación y Desarrollo, Universidad Peruana Cayetano Heredia, Lima, Peru; ^2^Division of Vaccine Discovery, La Jolla Institute for Allergy and Immunology, La Jolla, CA 92037, USA

## Abstract

Accurate measurement of B and T cell responses is a valuable tool to study autoimmunity, allergies, immunity to pathogens, and host-pathogen interactions and assist in the design and evaluation of T cell vaccines and immunotherapies. In this context, it is desirable to elucidate a method to select validated reference sets of epitopes to allow detection of T and B cells. However, the ever-growing information contained in the Immune Epitope Database (IEDB) and the differences in quality and subjects studied between epitope assays make this task complicated. In this study, we develop a novel method to automatically select reference epitope sets according to a categorization system employed by the IEDB. From the sets generated, three epitope sets (EBV, mycobacteria and dengue) were experimentally validated by detection of T cell reactivity *ex vivo* from human donors. Furthermore, a web application that will potentially be implemented in the IEDB was created to allow users the capacity to generate customized epitope sets.

## 1. Introduction

Adaptive immunity is based on the recognition of specific molecular structures, named epitopes, by either antibodies/B cell receptors or T cell receptors. Antibodies and B cell receptors bind a wide variety of structures, including proteins and carbohydrates. In the case of protein ligands, antibodies can recognize either a series of contiguous residues (linear epitopes) or a set of residues encoded in disparate regions of the protein sequence and brought together in the three dimensional structure of the protein ligand (discontinuous epitopes).

T cells recognize a complex between MHC molecules (named HLA in humans and H-2 in mouse) and, in most cases, a peptidic epitope of 8–16 residues in length [1, 2]. T cell responses are a key component of adaptive immunity. In concert with antibody responses, CD8 T cells, recognizing class I binding epitopes, and CD4 T cells, recognizing their class II counterparts, are key players in immunity to viruses and bacteria [3]. In the case of allergic reactions, CD4 T cell responses play a key role in pathogenesis both directly and indirectly through the regulation of antibody responses of the Ig E class [4].

Accurate measurement of B and T cell responses is a valuable tool to study autoimmunity, allergies, immunity to pathogens, and host-pathogen interactions and assist in the design and evaluation of T cell-based vaccines and immunotherapies [5–8]. Accordingly, a large number of studies have been devoted to defining B and T cell epitopes, a process that has been facilitated by an ever-increasing expansion and refinement of experimental methods.

As immune investigations proceed over time, many different epitopes from various organisms have been identified. Alternatively, large-scale epitope identification can reveal hundreds of potential epitopes [9–12]. The Immune Epitope Database (IEDB) [13, 14] is a freely available resource that serves as a repository of experimentally derived immune epitope information available in the peer-reviewed published literature, as well as from direct submission from NIH-NIAID funded large-scale epitope identification studies. The IEDB content covers a broad range of indications, to include infectious diseases (excluding HIV), allergies, transplantation, and autoimmune disease and a similarly broad range of hosts, including humans, nonhuman primates, mice, and livestock, amongst others. The database, as of October 2014, hosts experimental data related to 121,812 different peptidic epitope structures.

This large amount of information might, in some cases, pose a challenge for the identification and selection of appropriate sets of epitopes for use in specific contexts. Thus, it is clearly desirable, given the ever-growing body of information contained in the IEDB, to develop tools to enable the efficient generation of sets of validated reference epitopes for any antigenic source of interest.

While an epitope, according to classic definitions, is any structure capable of interacting with T and B cell receptors, in practice the consensus in the scientific community is that certain types of assays identify the most relevant and validated epitopes. In the context of antibody reactivity, by way of example, epitopes identified on the basis of X-ray structures of Ag/antibody complexes, biological activity, and* in vivo* assays or recognized by IgE in the case of allergens are considered more biologically relevant than linear epitopes recognized in ELISA assays and elicited by peptide immunization. In the case of T cell reactivity, again by way of example, multimer/tetramer staining assays or readouts based on ICS or ELISPOT assays, are preferred over older assay platforms such as Thymidine incorporation following multiple* in vitro* restimulations with peptides. These high quality assays have been selected based on our experience and judgment; however, in the web tool developed, the user will be capable of customizing it by his/her needs, that is, select only epitopes derived from neutralization or ELISA assays or any other desired selection.

Indeed, for many applications, it is desirable to study T cells* ex vivo*, without manipulation. This is because manipulations, such as* in vitro* expansion, are known to profoundly change the phenotype and characteristic of the T cells [15–17], thus questioning the physiological relevance of some of the experimental observations. However, direct* ex vivo* detection of human T cell responses is often difficult, largely because the immune response in any individual may target many different epitopes, and different individuals typically recognize unique epitope repertoires. Simultaneous use of many different epitopes as a pool might represent a powerful approach to detecting T cell responses, because even if the frequency of T cells recognizing each individual epitope may be below the limit of detection, a pool of a large number of epitopes (i.e., responses) might pass the limit of detection.

An important consideration in the definition of reference sets of epitopes is how to factor the number of individual donors or experiments in which a given structure is reported to elicit a positive response, and particularly if this validation is provided in multiple independent studies. For example, different studies often report on essentially the same epitope but utilize different nested, truncated, or frame-shifted version of the same sequence, leaving uncertainty on how to combine the data or which particular version of the epitope to select for testing. Clarification of a general approach for combining data from such disparate studies would greatly facilitate the generation of nonredundant sets of epitopes.

In the present study, we have attempted the definition of an automated process to generate reference sets of high quality epitopes for various disease indications. The resulting tool, made available to the scientific community, provides a standardized and reproducible platform to automatically extract and process relevant data from the IEDB without the need of complex analysis and judgment calls from the user. At the same time, the tool also offers flexibility to enable the end user to design sets meeting specific user-defined criteria. We have also analyzed the data currently available in the IEDB, to determine how many sets of pathogen or autoantigen specific epitopes could be identified on the basis of the data available to date.

## 2. Materials and Methods

### 2.1. Database, Processing, and Implementation

Epitope data was derived from the IEDB database as of October 2014. MySQL was used to run queries and directly work with the database itself. The web page application is written in PHP/HTML code with a MySQL connection that allows communication between the database and the user interface.

### 2.2. Ranking Scores on the Basis of Response Frequency and Assay Type

Two independent scoring systems were developed to allow ranking and sorting of the epitopes. The first was based on the type of assays used to characterize the epitopes and the second on the frequency by which each epitope was recognized.

Regarding the assay type scoring system for MHC class I or class II epitopes, in our selection we included epitopes defined by multimer/tetramer staining, ELISPOT, and ICS assays. We arbitrarily associate a numerical parameter value of 3, 2, and 1 to these assay types, respectively. Each of these assay types can be used in either an* ex vivo* or* in vitro* configuration. To provide that* ex vivo* assays are always ranked higher, we assigned an* ex vivo* configuration a value of 4, and an* in vitro* configuration a value of 1, and calculated a final assay score by multiplying the assay type and configuration values. In the case of B cell epitopes, X-ray structure, biological activity, and* in vivo* assays were assigned a score of 1, as well as epitopes that present IgE; for these epitopes, the effector origin was not taken in consideration. Since each epitope can be associated with multiple records, each describing different assays and thus with different assay scores, the highest assay score, reflecting the highest level of validation reached for that epitope, was selected and carried forward.

In terms of scoring each epitope on the basis of the frequency by which it was recognized, we utilized a previously described Response Frequency (RF) score [18]. The RF score is calculated as

(1) R−RN,

where *N* is the total of subjects tested and *R* is the number of positive responses. The square root is a correction factor, approximating one standard deviation for the number of responding donors. This gives a higher score to epitopes studied with larger sample sizes. In case there is no information of subjects tested, the epitope will be assigned *R* = 1, *N* = 1 if the assay outcome is positive and *R* = 0, *N* = 1 if it was negative. The RF score (RFS) also takes into account all data for a given epitope across all publications. We decided to use an older and simpler RF definition, as compared to the one used in [19], as it is more suitable to filter epitopes based on their RFS. As an example, consider an epitope 1 with positive responses (*P*) = 1 and total subjects (*T*) = 1; the new definition gives a RFS = 1 (0.04–1.00) whereas the older version gives an RFS = 0. In contrast with epitope 2 with *P* = 8 and *T* = 11, the new version gives RFS = 0.73 (0.43 : 0.92) and the older RFS = 0.47. In order for the RFS to reflect the difference between epitopes 1 and 2 in terms of the total number of subjects, the latter should be assigned a higher score to be used for ranking and filtering the data, which at the end benefits the user giving simpler and useful results.

Both the assay score and RF score are calculated for each epitope and provided in the results. This allows further ranking or selecting epitopes based on different thresholds for these criteria.

### 2.3. Generation of Consensus Sequences for Overlapping and Nested MHC Class I and Class II Epitopes

For MHC class I epitopes, it is generally observed that a length of about 8–11 residues is optimal for T cell recognition and use in assays. Because of the structure of the class I binding groove, distinct class I sequences typically represent unique epitopes, even if they are nested within a longer sequence that is also recognized by T cells. Accordingly, for the present study, we have not subjected class I epitopes of nested or overlapping character to further processing.

For MHC class II epitopes, however, optimal epitopes are usually longer than the minimal T cell recognized 9-mer core. In general, class II epitopes are optimally of 13–20 residues in length [1]. Peptides of varying length but that carry the same core may all be similarly active and/or recognized by the same T cell specificity. Thus, many of the epitope structures contained in the IEDB for class II epitopes are redundant, nested or largely overlapping. For this reason, it is desirable to devise strategies to reduce the complexity of class II epitope sets. Here, we developed a clustering algorithm to generate consensus sequences or cluster of epitopes, an illustration of such a process can be found in Table 1. In order to solve this problem, our approach first sorts the peptides based on their RF scores. Then, taking the highest ranked peptide as starting sequence, we move down the ranked list aligning the sequences to find nested or overlapping epitopes by at least 9 residues. For this approach, we only consider identical matches over the region of overlap and identical nested peptides; given this definition, mismatches will be treated as separate epitopes. When a nested peptide is found, we will keep only the larger peptide and calculate a new RF score using the sum of all responded and tested subjects per epitope in the cluster. For overlapping epitopes, a consensus epitope or cluster will be generated combining the sequences, if the cluster length is up to 20 residues. In these cases, the RF score will be calculated as a new RF score as in the nested case. For the assay type scoring system, the highest ranked assay and application of all the assays associated with the set of nested epitopes will be considered.

### 2.4. Human Study Subjects

In the case of donors with latent tuberculosis infection (LTBI), leukapheresis or whole unit blood samples from 10 adults were obtained from the University of California, San Diego, Antiviral Research Center (AVRC) clinic. Donors were classified as LTBI based on positive QuantiFERON-TB Gold In-Tube (Cellestis), as well as a physical exam and/or chest X-ray that was not consistent with active tuberculosis. Because Dengue virus (DENV) prevalence is low in the San Diego area, most LTBIs are DENV naïve.

To obtain DENV seropositive samples, anonymous blood donations from healthy adults were obtained by the National Blood Center, Ministry of Health, in the area of Colombo, Sri Lanka. Plasma of the associated donation was tested for serology using the flow-based U937+DC-SIGN neutralization assay (conducted at the University of North Carolina, Chapel Hill) as previously described [11, 20]. Because TB prevalence is low in the Colombo area, most DENV seropositive donors can be assumed to be TB negative.

All Samples were collected and used following guidelines from the Institutional Review Boards (IRB) of LJI and the Medical Faculty, University of Colombo (serving as National Institutes of Health-approved IRB for Genetech Research Institute).

### 2.5. Peptides and PBMC Isolation

15-mer peptides were synthesized as crude material on a small (1 mg) scale by Mimotopes (Victoria, Australia) and/or A and A (San Diego). PBMCs were purified by density gradient centrifugation (Ficoll-Hypaque, Amersham Biosciences) from 100 mL of leukapheresis sample or 450 mL of whole blood, according to manufacturer's instructions. Cells were cryopreserved in liquid nitrogen suspended in fetal bovine serum (Gemini Bio-products) containing 10% dimethyl sulfoxide.

### 2.6. Intracellular Cytokine Staining (ICS)

PBMCs (2 × 10^6^ cells/well) were incubated with peptide pools (1 *μ*g/mL) for 2 hrs. Brefeldin A (1 *μ*g/mL) (BD Bioscience) was added to the mixture and incubated for another 4 hours (i.e., a total of 6 hrs with peptide). Cells were then washed and stained for cell surface markers using anti-CD3-Alexa Fluor 700 (UCHT1), anti-CD8-V500 (RPA-T8) (both from BD Biosciences), anti-CD4-APC EFluor780 (RPA-T4), anti-CD45RA-EFluor450 (HI100), and anti-CCR7(CD197)-PerCPCy5.5 (G043H7) (all three from Affymetrix eBioscience) for 30 mins on ice. Cells were then washed, fixed with 4% paraformaldehyde, blocked with human sera, and stained for intracellular IFN*γ* using anti-IFN*γ*-FITC (4S.B3, Affymetrix eBiosciences). Samples were acquired on a BD LSR II flow cytometer. The frequency of cells responding to the TB/DENV-specific peptides was quantified by determining the total number of gated subset+ and cytokine+ cells and background values subtracted (as determined from the medium alone control) using FlowJo software (Tree Star).

## 3. Results and Discussion

### 3.1. Filtering Datasets to Select Human Peptidic Epitopes of Appropriate Size, Defined Restriction, and Assay Type

As a preliminary step towards deriving sets of reference epitopes associated with preferred validated assays, we processed the data contained in the IEDB relating to T cell epitopes. As of October 2014, a total of 28370 epitopes are associated with positive results in at least one T cell assay.

**Figure 1:**
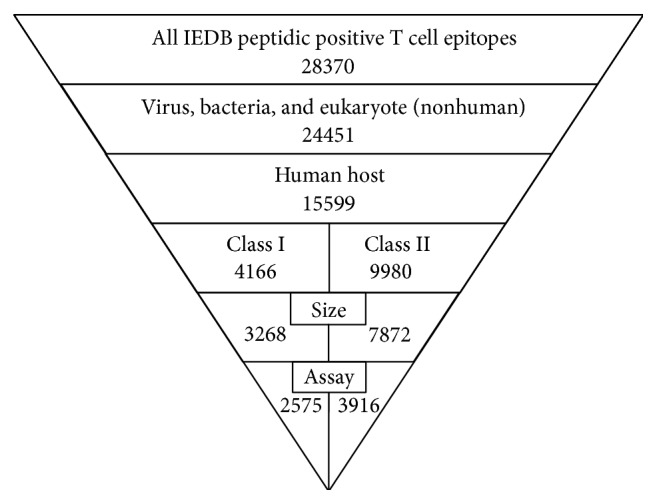
Diagram of the filtering steps towards the generation of the validated sets of epitopes, including the number of epitopes found in each step.

As an example of filtering strategies, we first considered only peptidic epitopes associated with infectious agents and allergies (Figure 1) and initially focused on bacteria, viruses, and nonhuman eukaryotes as epitope sources. Next, we only considered data in which humans were the host of the immune response and for which data was available to allow specifically characterizing responses as either class I or class II restricted. In the case of class I, we further considered only epitopes of 8–11 residues, and in the case of class II, only peptides of 13–20 residues were considered.

The next step in our process was to filter the results further by selecting epitopes that have been tested in “high quality” assays. This is possible because the IEDB curates the specific assays that are used to define and characterize the specific epitopes reported in the literature or provided to the database by direct submission. While obviously any desired assay set could be used, here we selected for inclusion the multimer/tetramer staining, ELISPOT and ICS assays. This assay-based filtering resulted in a final total of 6345 epitopes, 2512 and 3833 for class I and class II epitopes, respectively (Figure 1), representing about 20% of the initial 28370 epitopes.

### 3.2. Identification of Epitope Categories Supported by Current IEDB Data

We surveyed the epitope data in the IEDB in terms of the species and antigens of provenance (epitope sources). For this purpose we adapted the categorization adopted by Seymour et al. [21]. The relative population of these categories in the IEDB as of the 2009 date was discussed by Davies et al. [22].

In Table 2, we list 43 categories for which at least ten class I or class II epitopes were identified for viruses/bacteria (Table 2(a)), nonhuman eukaryotes (Table 2(b)), and autoantigens (Table 2(c)). The data were further classified according to the source organism of the epitope. The number of epitopes contained in each category is also listed in Table 2. The table also lists B cell epitopes as identified and discussed further below.

### 3.3. Definition of Epitope Tables

As a result of the processes described above, we generated sets of epitopes for the various categories. As an example, Table 3 details the 21 epitopes defined for the Parvoviridae human class I category. In addition to the epitope sequence, source organism and protein, and MHC restriction, the IEDB Epitope ID is provided to facilitate retrieval of additional information from the IEDB pertaining to the epitope. Finally, the Response Frequency, Assay Score, the types of assay, and effector origin (*ex vivo* or* in vitro*), utilized to derive the Assay Score, are also given. All epitope tables from the categories shown in Table 2 can be found in the supplemental file “Epitope Tables.zip” in the Supplementary Material available online at http://dx.doi.org/10.1155/2015/763461.

### 3.4. Expanding the Tool to Address Autoimmune Epitopes, B Cell Epitopes, and Murine Epitopes

Having established the conceptual framework for selection of epitope sets, we next expanded our applicability. Autoimmune epitopes are identified by the fact that both source antigen and host organism are the same (e.g., both the T cells and the epitope are originated from a human source). As listed in Table 2(c), all autoimmunity epitope categories relevant to a given disease are condensed into a single category. A protein tree functionality available on the IEDB [14] and a list of the most common antigens associated with each disease were used to extract autoimmune epitopes. The identified categories for which at least ten class I or class II epitopes were available are listed in Table 2(c), along with the number of epitopes contained in each category.

We considered expanding the scope of the study to also select epitopes recognized by species other than humans. In this case, the second most frequently represented host species is mouse. Accordingly, an option was created in the web application (next section) to allow selection of murine epitopes. The number of murine epitopes identified is listed as a separate column in Table 2.

Finally, we also expanded our analysis to allow selection of B cell/antibody epitopes. In this case, we set a 5 to 20 residue size window and initially selected X-ray structure, biological activity, and* in vivo* assays as most biologically relevant. In addition, we included induction of IgE subclass responses, as this type is most relevant in the context of allergic diseases. The number of B cell epitopes identified accordingly is also listed as a separate column in Table 2.

### 3.5. Implementation of a Web Application to Automatically Generate Epitope Sets

Finally, we developed a tool, which will be hosted by the IEDB as an additional link in the search results page and will be part of the next IEDB update release in fall 2015. This tool allows generation of specific epitope sets following the default criteria described above but also allows users to customize the generation of novel sets.

**Figure 2:**
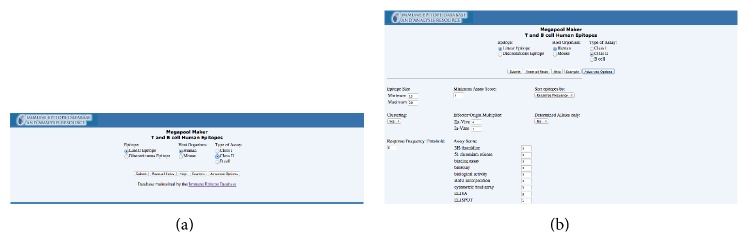
(a) Web application main page interface. (b) Web application “advanced options” page interface.

A sample screen shot of the main interface is shown in Figure 2(a). The main interface automatically obtains query results from IEDB searches and adds them to the query parameters of the tool. There, the user may choose to generate sets of class I, class II, antibody linear, or antibody discontinuous epitopes, relating to either human or mouse as the host organism from which the epitopes are derived. Because of the flexibility of the design, additional host organisms may be included, provided sufficient data becomes available in the literature. The user is offered results obtained with the default settings.

An “advanced options” webpage can be accessed from the main page, and a sample screen shot of this option is shown in Figure 2(b). In the advanced options, the user can set their custom RF score threshold and Assay score threshold, rank the results using the RF score or Assay Score, include the clustering tool, filtering by determined alleles (known MHC allele), and set the minimum and maximum size if the epitopes. Also, a number of additional assays, generally considered as being less rigorous, are provided, allowing the user to set custom parameters. Every field in the advance options dynamically changes to fit the default values for a given query. For example, if the user selects MHC class II epitopes, the web tool will set the clustering field to “yes” and the epitope size to 13–20.

### 3.6. Selection and Synthesis of Peptide Sets for Experimental Validation

To experimentally validate the usefulness of the tool we decided to synthesize some of the actual peptide sets identified by the tool and experimentally test them for recognition by human T cell responses. One of the main challenges for testing large pools for T cell recognition is that* ex vivo* T cells assays require relatively substantial amounts of each epitope (in the 1–0.1 microgram/mL range), compounded by the fact that peptide solubility in solvents most widely applicable to preparing peptide stock solutions, such as DMSO, is usually limited to about 20–40 mg/mL. Since solvents like DMSO are toxic in cellular assays at concentrations above 0.5%, this would seem to effectively limit the number of peptides that can be safely included in a pool to about 20.

However, in many cases the solubility of one peptide is not drastically influenced by the presence of other peptides (especially if the sequences, isolectric point, and general solubility are different). For this reason, we predicted that it might be possible to make pools of peptides already dissolved in a solvent like DMSO, mix the solutions, and relyophilize the pool of pools. Indeed, we routinely find these “sequentially lyophilized” pools, once resuspended, to be much more soluble than the individual components.

Accordingly, we synthetized a set of 207 EBV human CD8/class I epitopes, identified by the default setting described above (Supplemental Table 1A). In addition, we also synthetized a set of 92 CD8/class I epitopes derived from DENV virus, obtained by selecting only peptides with RF > = 0.01 (Supplemental Table 1B), and a set of 86 epitopes CD4/class II epitopes derived from* Mycobacterium tuberculosis*, based on an RF score > = 0.1 and being recognized* ex vivo* (Supplemental Table 1C).

### 3.7. Experimental Validation of the Use of Reference Epitope Sets to Detect T Cell Reactivity* Ex Vivo*

Peptides corresponding to these three sets of epitopes were pooled and tested with human PBMC as a source of T cells. For these experiments we selected PBMC from 5 individuals infected with DENV virus and likely uninfected with TB (see methods for details) and PBMC from 5 LTBI individuals and likely uninfected with DENV. Because of the high incidence of EBV infection worldwide [24], we assumed that most if not all individuals tested would be latently infected with EBV.

**Figure 3:**
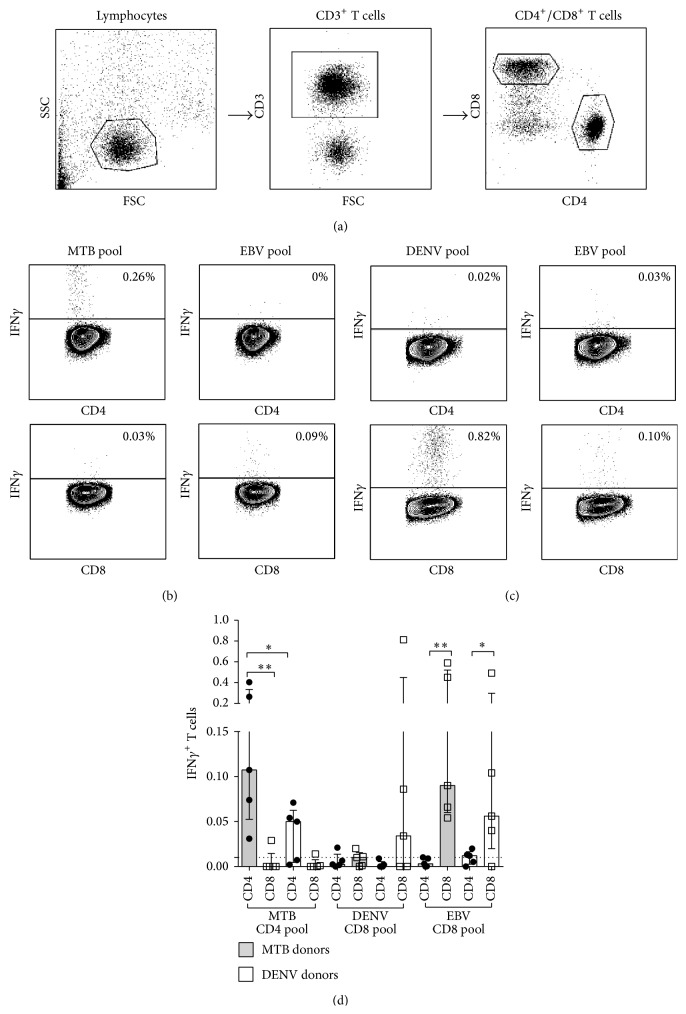
Predicted epitope pools induce a detectable* ex vivo* cytokine response. PBMC from donors with LTBI (MTB donor) or previously exposed to DENV were stimulated with their respective epitope pools and an EBV epitope pool. After* ex vivo *stimulation, the IFN*γ* response was measured by ICS using flow cytometry. Gating strategy (a) and representative plots of the responses for both CD4^+^ and CD8^+^ T cells are shown for a MTB donor (b) and DENV donor (c). (d) The ICS IFN*γ* responses induced by each epitope pool for all donors tested are summarized (*n* = 10). Statistical difference was determined using a one-tailed Mann-Whitney test. (^∗^ *P* ≤ 0.05; ^∗∗^ *P* ≤ 0.01).

PBMC were stimulated with the DENV CD8 pool, MTB CD4 pool, and EBV CD8 pool. After* ex vivo *stimulation, the IFN*γ* response was measured by ICS (Figures 3(a)–3(d)). The gating strategy for these experiments is presented in Figure 3(a). Representative plots of the responses for both CD4^+^ and CD8^+^ T cells are shown for a LTBI donor (Figure 3(b)) and a DENV donor (Figure 3(c)). The IFN*γ* responses induced by each epitope pool in the relevant CD4/CD8 compartments for all donors tested are summarized in Figure 3(d). In the case of the MTB CD4 pool,* ex vivo* CD4 but not CD8 T cell responses were seen in the LTBI (*P* = 0.004) and DENV donors. The reactivity was lower in the DENV donors than in the LTBI donors (*P* = 0.03). This lower, but detectable, reactivity is explained by the fact that the DENV seropositive individuals are vaccinated against MTB with* M. bovis* BCG and are likely exposed to nontuberculous mycobacteria [25]. In the case of the DENV CD8 pool, CD8 T cell responses, but not CD4, were detected in 3 out of 5 DENV donors. This* ex vivo* reactivity in 3 out of 5 donors is in line with what was detected in previous studies [11]. As expected, no CD4 responses were seen to the CD8 DENV pool, as also low CD8 and CD4 T cell reactivity was noted in the MTB donors with the DENV CD8 pool. Finally, as also expected, in both cohorts reactivity was detected against the EBV CD8 pool in CD8 but not in CD4 T cells (*P* = 0.004 for LTBI and *P* = 0.05 for DENV).

## 4. Conclusions

We devised a strategy that allows automatically filtering datasets to select epitopes of appropriate size, defined restriction, and assay type for use in characterizing responses to specific indications. While querying the IEDB database can also generate these sets, a certain degree of complexity in the queries and the setting of multiple parameters would be necessary. In our application, the epitope sets are automatically generated, while the user is still enabled to change the default settings to generate validated epitope sets matching specific criteria.

We further identified which epitope categories are supported by current IEDB data, and found that reference epitope sets could be produced for 43 categories with data currently available in the IEDB. The number of such categories, broadly based on previous epitope classification work [21], is undoubtedly destined to grow, allowing an ever more comprehensive study of immune responses in a broad variety of experimental systems. To further illustrate the broad applicability of the approach, we also extended our work to address autoimmune epitopes, B cell epitopes, and murine epitopes.

While these actual epitope sets are provided as tables within the paper, we implemented a web application to automatically generate epitope sets, based on the fact that the IEDB content is rapidly growing and new epitopes are added to the IEDB in each of its biweekly updates. We plan to continuously gather feedback on this web application from the scientific community, and to implement changes and modifications through the main IEDB website [14].

Finally, to illustrate applicability in an actual experimental setting, we selected and synthesized peptide sets corresponding to EBV, DENV, and MTB epitopes. These epitope sets were used to measure immune reactivity in human cells. The experimental testing of these epitope sets demonstrated the applicability of these sets as a valuable resource to allow detection of T cell responses* ex vivo*. As such, these sets of epitopes represent potentially valuable resources for diagnostic purposes, as well as for further detailed characterization of the immune response to specific targets of immunological interest.

## Supplementary Material

Supplemental tables 1A, 1B and 1C contain the epitope sets used in the experimental validation of the EBV, DENV and *Mycobacterium tuberculosis* sets. The compressed file “Epitope Tables” contains the epitope tables for mouse (H-2) and human (HLA) of T cell class I (H-2 I and HLA I) and class II (H-2 II and HLA II). In the case of B cell epitopes, they are divided into discontinuous (Mouse B cell Discont and Human B cell Discont) and linear (Mouse B cell Linear and Human B cell Linear). For each of these directories, the epitope tables of each category (Table 2) are found inside.”

## Figures and Tables

**Table:** (a) Epitopes before being processed by the clustering tool; epitopes forming a potential consensus sequence or cluster are in bold

Epitope/cluster	RF score	Assay score
MLVLLVAVLVTAVYAFVHA	0.67	8
**IQGNVTIHSLLDEGK**	**0.66**	**8**
VPSPSMGRDIKVQFQSGGAN	0.65	12
NVTSIHSLLDEGKPT	0.63	12
**QGNVTIHSLLDEGKPT**	**0.59**	**12**
AQAAVVRFQEAANKQKQELD	0.47	12
**GNVTSIHSLLDEG**	**0.46**	**4**
FAGIEAAASAIQGNV	0.42	12

**Table:** (b) The cluster generated by combining the sequences and associated information is in bold

Epitope/cluster	RF score	Assay score
MLVLLVAVLVTAVYAFVHA	0.67	8
VPSPSMGRDIKVQFQSGGAN	0.65	12
**IQGNVTIHSLLDEGKPT**	**0.57**	**12**
AQAAVVRFQEAANKQKQELD	0.47	12
FAGIEAAASAIQGNV	0.42	12

**(a):** 

	Class I		Class II		B cell			
					Linear		Discontinuous	
	HLA	H-2	HLA	H-2	Human	Mouse	Human	Mouse
Virus								
ssRNA (−) strand virus								
H1N1 subtype influenza A	41	77	207	206		15	11	27
H3N2 subtype influenza A	18	18	92	17		11	22	81
Other influenza A subtypes (not H3N2 or H1N1)	116	43	179	25		43	16	81
Influenza B/C			13					36
Paramyxoviridae (respiratory syncytial virus, measles, mumps)	47	80	28	27		33		71
Hantavirus	14							
ssRNA (+) strand virus								
Dengue virus	432	116	58	139		24	29	97
Hepatitis C virus	405	65	241	20	53	32	27	12
West Nile virus	33		99	103				24
Yellow fever	18	34	94	118				
Japanese encephalitis virus			33					
Picornaviridae (coxsackie, hepatitis A)	14	12		52		77		94
Coronaviruses	22	38	38	25	23	28		27
Retrotranscribing virus								
Hepatitis B virus	59	72	38	18		13		
*Deltaretrovirus* (HTLV)	25							
dsDNA virus								
Adenoviruses	13		45			10		
Alphaherpesvirinae (human herpesvirus 1/2, Varicellovirus)	91	52	35	13		32		32
Betaherpesvirinae (CMV, human herpesvirus 5, roseolovirus, murid herpesvirus)	204	48	141	20				
Gammaherpesvirinae (Epstein-Barr virus, *Rhadinovirus*, human herpesvirus 4)	237	63	59					
Papillomaviridae (human papillomavirus)	80	44	72			30		
Poxviridae (vaccinia, pox)	228	343	76	30				
Polyomavirus (Simian vacuolating virus)	31	14						
Parvoviridae	21		24		10			
Bacteria								
Actinobacteria/proteobacteria								
Alphaproteobacteria (Rhizobiales, *Rickettsia*, and *Anaplasmas*)			31					
Betaproteobacteria (*Neisseria*, *Bordetella*, and *Burkholderia*)			324	158		33		
Mycobacterium	129	33	478	33				11
Firmicutes/other bacteria								
Chlamydiales (chlamydia)	15	38				37		
Clostridiales			70					
Other Bacilli (anthracis, cereus, *Geobacillus*, and *Enterococcus*)			106			19		

**(b):** 

	Class I		Class II		B cell			
					Linear		Discontinuous	
	HLA	H-2	HLA	H-2	Human	Mouse	Human	Mouse
Alveolata								
Plasmodium *(P. falciparum*, * P. vinckeia*, and *P. yoelli*)	64	29	186	33	16	49		
Euglenozoa								
Trypanosomatidae (*Trypanosoma*, *Leishmania*)	91	30		14				
Fungi								
*Aspergillus *			50	50	73			
Other fungi			77		54	11		
Plants								
Fabaceae (peas, soybean, peanut family)			17		419			
Betulaceae (birch family)			30		24			
Cupressaceae (cypress, cedar family)			21		22			
Gluten, coeliac Disease^1^			23		245	36		
Timothy-grass			474		19			
Other grass			98		124			
Amaranthaceae			20					
Animals								
Insects			67		23			
Arachnid			97		33	13	14	
Mammals		119	41	69	707	80		15

^1^Only epitopes derived from gliadin protein and high molecular weight glutenin [23] were included.

**(c):** 

	Class I		Class II		B cell			
					Linear		Discontinuous	
	HLA	H-2	HLA	H-2	Human	Mouse	Human	Mouse
Rheumatoid arthritis	27		11					
Diabetes	73	76	46	17				
Multiple sclerosis			11		13			

**Table 3:** Parvoviridae virus validated epitope set downloaded from the web tool.

Epitope/cluster	Epitope ID	Source organism	Source protein	MHC restriction	RF score	Assay score	Assay type	Effector origin
FYTPLADQF	18474	Human parvovirus B19	Noncapsid protein NS-1	HLA-A^*^24:02	0.51	12	Multimer/tetramer, 51 chromium, ELISPOT	Direct *ex vivo*, cell line/clone
GLCPHCINV	20786	Human parvovirus B19	Noncapsid protein NS-1	HLA-A^*^02:01, HLA-A2	0.46	4	ELISPOT, 51 chromium	Direct *ex vivo*
QPTRVDQKM	51981	Human parvovirus B19	Noncapsid protein NS-1	HLA-B35	0.29	3	ELISPOT, 51 chromium, multimer/tetramer	Cell line/clone
LLHTDFEQV	37397	Human parvovirus B19	Noncapsid protein NS-1	HLA-A^*^02:01, HLA-A2	0.21	4	ELISPOT, 51 chromium	Direct *ex vivo*
TAKSRVHPL	62900	Human parvovirus B19	Viral protein 2	HLA-B8	0.12	4	ELISPOT, 51 chromium	Direct *ex vivo*
TEADVQQWL	63285	Human parvovirus B19	Noncapsid protein NS-1	HLA-B40	0.1	4	ELISPOT, 51 chromium	Direct *ex vivo*
SSHSGSFQI	61077	Human parvovirus B19	Noncapsid protein NS-1	HLA-Class I	0	4	ELISPOT, 51 chromium	Direct *ex vivo*
SESSFFNLI	57628	Human parvovirus B19	Noncapsid protein NS-1	HLA-B40	0	4	ELISPOT	Direct *ex vivo*
VQQWLTWCN	70634	Human parvovirus B19	Noncapsid protein NS-1	HLA-Class I	0	4	51 chromium, ELISPOT	Direct *ex vivo*
VPQYGYLTL	70458	Adeno- associated virus - 2	Major coat protein VP1	HLA-B^*^07:02	0	2	ICS, biological activity, ELISA	Short term restimulated
SALKLAIYKA	56861	Human parvovirus B19	Noncapsid protein NS-1	HLA-Class I	0	8	RNA/DNA detection, ICS	Direct *ex vivo*
TEADVQQWLTW	63286	Human parvovirus B19	Non-capsid protein NS-1	HLA-B44	0	4	ELISPOT	Direct *ex vivo*
QSALKLAIYK	52287	Human parvovirus B19	Noncapsid protein NS-1	HLA-Class I	0	8	ICS	Direct *ex vivo*
IDTCISATFR	25677	Human parvovirus B19	Noncapsid protein NS-1	HLA-Class I	0	4	ELISPOT	Direct *ex vivo*
HAKALKERMV	23542	Human parvovirus B19	Noncapsid protein NS-1	HLA-Class I	0	4	ELISPOT	Direct *ex vivo*
GLFNNVLYH	20861	Human parvovirus B19	Noncapsid protein NS-1	HLA-Class I	0	4	51 chromium, ELISPOT	Direct *ex vivo*
LHTDFEQVM	36432	Human parvovirus B19	Noncapsid protein NS-1	HLA-Class I	0	4	ELISPOT, 51 chromium	Direct *ex vivo*
LLHTDFEQVM	37398	Human parvovirus B19	Noncapsid protein NS-1	HLA-A^*^02:01	0	8	ICS	Direct *ex vivo*
GLCPHCINVG	20787	Human parvovirus B19	Noncapsid protein NS-1	HLA-Class I	0	8	ICS, RNA/DNA detection	Direct *ex vivo*
EADVQQWLT	11014	Human parvovirus B19	Noncapsid protein NS-1	HLA-Class I	0	4	ELISPOT, 51 chromium	Direct *ex vivo*
RMTENIVEV	145986	Human parvovirus 4	ORF1	HLA-A^*^02:01	0	12	Multimer/tetramer, ICS	Short term restimulated, direct *ex vivo*
